# Avoidance of simultaneous patch use in Japanese large-footed bats

**DOI:** 10.1371/journal.pone.0343485

**Published:** 2026-06-30

**Authors:** Emyo Fujioka, Masashi Shiraishi, Tamao Hirao, Yui Onishi, Dai Fukui, Shizuko Hiryu

**Affiliations:** 1 Organization for Research Initiatives and Development, Doshisha University, Kyotanabe, Kyoto, Japan; 2 Graduate School of Information Sciences, Hiroshima City University, Hiroshima, Japan; 3 Faculty of Life and Medical Sciences, Doshisha University, Kyotanabe, Kyoto, Japan; 4 The University of Tokyo Fuji Iyashinomori Woodland Study Center, Graduate School of Agricultural and Life Sciences, The University of Tokyo, Minamitsuru-gun, Yamanashi, Japan; National Museums of Kenya, KENYA

## Abstract

Group foraging can enhance prey detection, but depending on resource availability, it may also generate conflicts among conspecifics. To understand how animals balance these benefits and costs, foraging performance must be evaluated together with inter-individual interactions. However, under fully natural conditions, it remains challenging to quantify both simultaneously. Here, we investigated how individual foraging efficiency and pairwise interactions are shaped when more than one individual simultaneously exploit the same foraging patch, using the Japanese large-footed bat (*Myotis macrodactylus*) as a model system. We monitored an entire pond functioning as a natural foraging patch using two thermal cameras and an eight-channel microphone array, and reconstructed the arrival, prey-attack, and exit times of individual bats. Using a Poisson generalized linear mixed model (GLMM), we found that prey-attack rates were approximately 25% lower during paired flights than during solitary flights. We then constructed a null model in which arrival, attack, and departure events followed independent Poisson processes parameterized from the empirical data. Compared with null-model predictions, both the total duration and the duration of individual paired flights in the empirical data were significantly shorter, indicating that bats limited the time spent co-using the same patch relative to solitary foraging. In addition, the probability that the first exiting individual was the one that arrived earlier or later did not deviate from chance levels, providing no evidence for a prior residence advantage. Together, these results indicate that co-use duration was shorter than expected under the null model regardless of arrival order and was accompanied by a reduced prey-attack rate during simultaneous patch use. These findings suggest that bats tend to avoid prolonged shared patch use, which may help maintain prey-attack efficiency. Our findings highlight bats as an excellent model system for non-invasively linking individual behavior and foraging performance via echolocation, and for elucidating the dynamics of foraging behavior and sensory interference in the wild.

## Introduction

Foraging behavior is one of the most fundamental activities in animals and directly affects survival and fitness [1,2]. Many predatory species do not forage exclusively alone but instead adopt strategies involving temporary or permanent group formation (e.g., fish [3,4]; ants [5,6]; birds [7,8]). Such strategies confer multiple advantages, including an expansion of individual perceptual ranges that enhances prey-detection capabilities [9,10], improved access to temporally and spatially ephemeral food patches [11,12], and increased foraging success through information transfer among group members, which facilitates efficient food searching and hunting [13,14]. At the same time, group foraging entails costs, such as intensified intraspecific competition and interference arising from resource sharing [15,16], as well as reductions in per capita food intake [17,18]. In patchy environments, the profitability of a feeding site depends not only on prey abundance but also on the density of competing individuals. Optimal foraging and patch-use theories predict that animals should leave a patch and redistribute to alternative sites when local competition or interference reduces foraging payoff [1,19,20]. In group-foraging animals, such interference can arise not only through direct competition for prey but also through sensory interference and spatial overlap during prey capture attempts [21,22]. Although these trade-offs have been demonstrated in numerous studies at the individual level (e.g., [10,12]) and under controlled conditions (e.g., [14,17]), quantitatively observing the foraging behavior and interactions of multiple freely moving individuals in fully natural environments, and understanding how predation is actually carried out under such conditions, remain extremely challenging.

Bats rely on echolocation for navigation and prey detection in darkness [23,24]. Echolocation calls contain rich information about the direction of attention and the behavioral state of an individual. For example, feeding buzzes emitted immediately prior to prey capture indicate when a bat is capturing target prey, and the interval of ultrasonic emissions allows precise identification of the timing at which prey-approach behavior is initiated. Thus, in bats, echolocation calls enable high-resolution quantification of foraging behavior under fully natural conditions [25,26]. In particular, insectivorous bats that forage over water use echolocation to detect emerging aquatic insects near the water surface and capture them while trawling over ponds and streams [27,28]. This behavioral specialization facilitates fixed-site observations of a foraging patch, making this species an excellent wild model for quantitatively analyzing foraging behavior and inter-individual interactions based on acoustic recordings.

A distinctive feature of inter-individual interactions during bat foraging is that echolocation calls and their echoes are audible not only to the emitting individual but also to nearby conspecifics. Listening to the calls of others can yield both benefits and costs during foraging. For example, it has been reported that bats—including both conspecifics and heterospecifics—eavesdrop on the echolocation calls of others to obtain profitable information about foraging sites [29–32]. At the same time, calls emitted by nearby individuals can interfere with a bat’s own echoes, potentially degrading echolocation performance and reducing foraging efficiency. Indeed, recent studies have highlighted a trade-off in group foraging, whereby foraging with a small number of conspecifics can facilitate prey detection, whereas the presence of too many individuals can instead reduce efficiency [33]. Radio-tracking studies have suggested that *Myotis daubentonii*, an ecologically similar congener that forages over water surfaces, exhibits temporal segregation in the use of foraging areas, resulting in limited simultaneous use by multiple individuals [34]. Considering these opposing benefits and costs, understanding bat foraging strategies when multiple individuals occupy the same foraging patch requires quantitative evaluation not only of individual behavior but also of its relationship with local prey resources.

In Japanese large-footed bats (*Myotis macrodactylus*), aquatic insects targeted during foraging emerge at the water surface and exit shortly thereafter, such that competition is expected among individuals attempting to exploit prey immediately after emergence. Accordingly, analyses of foraging behavior in this species provide an opportunity to reveal dynamic inter-individual interactions and strategic adjustments within a shared foraging patch. In this study, we monitored an entire pond as a natural foraging site and examined the foraging behavior of Japanese large-footed bats that arrived sequentially at the site, with particular attention to periods in which more than one individual was co-using the site simultaneously. At the focal pond, both solitary foraging events and situations in which additional bats arrived and foraged simultaneously were observed. In echolocation-based foraging targeting prey at the water surface, acoustic interference arising from reflections off the water surface is expected to occur—an effect that is absent during foraging in featureless three-dimensional open space. Consequently, when the foraging patch is spatially restricted, sharing the patch with conspecifics is predicted to impose substantial echolocation-related costs on Japanese large-footed bats. Based on this reasoning, we hypothesized that the presence of other bats within the same foraging patch reduces foraging efficiency and that individuals adopt behavioral strategies to avoid co-using the patch with conspecifics.

To test this hypothesis, we deployed wide–field video cameras and a microphone array configured to cover the entire study pond, allowing us to record the arrival and departure times of successive bats as well as their prey-capture behavior within the foraging patch. We further modeled bat arrival, prey-attack, and exit events at the patch as Poisson processes and estimated their occurrence rates from the empirical data to construct a null model that excludes inter-individual interactions. By comparing the empirical data with this null model, we evaluated whether bats adjust their behavior in response to the presence of conspecifics when co-using the same patch—that is, whether patch staying times deviate from expectations under a simple probabilistic process. The primary objective of this study was to investigate how Japanese large-footed bats adjust their behavior under natural conditions when more than one individual simultaneously exploits the same foraging patch and to clarify the relationship between such behavioral adjustments and foraging efficiency. To further address this objective, we additionally analyzed which individual was more likely to leave first during simultaneous multi-individual foraging bouts, providing complementary insights into inter-individual interactions during bat foraging.

## Materials and methods

### Target species and study site

Target species of this study is Japanese large-footed bats, *Myotis macrodactylus.* This bat species performs echolocation using frequency-modulated calls [28,35]. When attacking insect prey, individuals emit a feeding buzz, during which the call frequency decreases immediately prior to the attack [28,36]. This indicates that the timing of prey-attack events can be identified from the recorded echolocation sounds. We defined foraging efficiency as the frequency of attack attempts on prey, that is, the encounter rate with prey.

**Fig 1:**
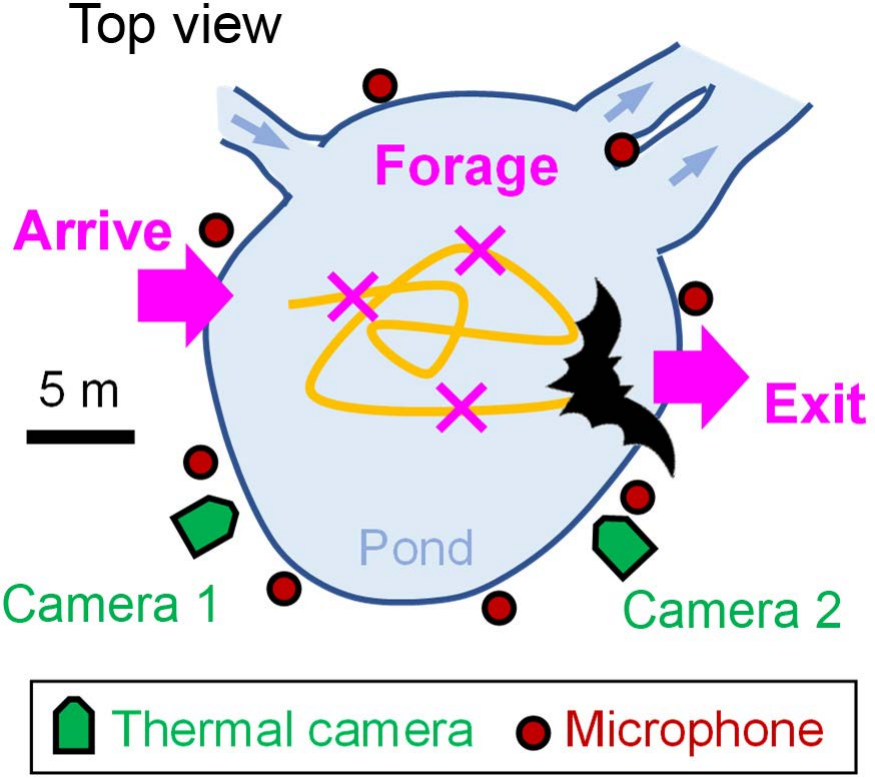
Schematic diagram of the study site and measurement system. Blue arrows show the direction of the stream.

The study site was a single pond (approximately 20 × 20 m) located within the Tomakomai Experimental Forest of Hokkaido University (Fig 1), which is one of several ponds downstream from this pond distributed along Horonai stream within the forest. At this pond, *M. macrodactylus* have been observed foraging just above the water surface alone or in the presence of conspecifics [36]. Field recordings were conducted on three nights (12, 16, and 17 September 2024). Data collection began shortly after sunset (approximately 17:30), when the first bat appeared, and continued for approximately one hour each night.

Although the study site is designated as a wildlife reserve by the local government, no specific permits were required for this study because it was a non-invasive observational study that did not involve any endangered or protected species, animal capture, or habitat disturbance. The observations at the pond were conducted with permission from the forest administration.

### Video and acoustic recording and data analysis

For video recordings, two FLIR A65 thermal video cameras (field of view: 90°, frame rate: 30 fps; Teledyne FLIR LLC, Oregon, USA) were used to record bat arrivals at and exits from the pond as monochrome video files on a personal computer (Fig 1). The target bat species can be detected at a range of approximately 20 m from the camera. The recordings from the two cameras were temporally synchronized by clapping hands within the overlapping fields of view of both cameras. From the recorded videos, the arrival and exit times (hh:mm:ss) of successive bats were extracted using video playback software, Media Player Classic (ver. 6.4.9.0) and ImageJ (ver. 1.54g). Inter-arrival intervals and patch residence times were then calculated for each bat.

For acoustic recordings, eight ultrasonic-band MEMS microphones (custom-made, based on the SPU0410LR5H; Knowles) were deployed surrounding the pond (Fig 1). This configuration enabled comprehensive recording of all vocalizations produced within the pond, including feeding buzzes emitted by individual bats. Acoustic signals captured by the microphones were recorded on a personal computer via a data acquisition device (USB-6356; National Instruments, Inc., USA) at a sampling rate of 500 kHz. Audio and video recordings were synchronized using the handclap sounds that were also used for video synchronization.

Based on our previous synchronized video–acoustic recordings [36], echolocation calls of this species can typically be detected at distances of up to approximately 20–30 m from a microphone. However, the detectable range varies depending on the emission direction of the bat and the echolocation phase (e.g., search, approach, or terminal phase). In the present study, eight microphones were arranged surrounding the pond. Because the pond was approximately 20 m in diameter, this microphone configuration was considered sufficient to comprehensively record echolocation calls produced by bats visiting the focal pond.

The timing of prey-attack events was determined from the acoustic data by visually identifying the terminal portion of feeding buzzes in which call frequency decreased using Adobe Audition 24.2.0.83, with a temporal resolution of 0.1 s. When no frequency decrease was observed, the event was recorded as a non-attack [36]. In this study, situations in which only a single bat was present within the foraging area were defined as the single-bat context, whereas situations involving two or more bats were defined as the multiple-bat context. In the multiple-bat context, the individual flying near the microphone that recorded the feeding buzz was determined to be the emitter of the feeding buzz. When it is hard to identify the feeding-buzz emitter using such snapshot of video and acoustic recordings because inter-individual distance was too small, the emitter was identified using time differences of arrival (TDOA) in the microphone array. Specifically, the relative spatial positions of the bats were determined from the temporal patterns of TDOA in acoustic sequences containing a feeding buzz recorded by the microphones near the bats. Using our microphone array system, the localization accuracy of sound sources estimated from FM echolocation pulses emitted by bats was approximately 20–30 cm at most [37]. Because the inter-individual distance between bats rarely became smaller than this range in our recordings, we were able to continuously distinguish and track individual bats throughout the observations. Then, the bat that emitted feeding buzz was identified by comparing the acoustically determined relative positions with the bats’ flight positions observed in the synchronized video recordings.

### Null model construction and statistical analysis

We constructed a stochastic behavioral simulation model in MATLAB (MathWorks, USA), in which three events at the foraging site—arrival, prey attack, and exit—were treated as independent Poisson processes. This model was positioned as a null model that does not incorporate inter-individual interactions, and the event rates (occurrence probabilities per unit time) for all processes were derived entirely from the empirical data obtained in this study. Specifically, the arrival rate was defined as the number of bats arriving at the foraging site per unit time. The prey-attack rate was estimated separately for the single-bat and multiple-bat contexts by modeling the number of prey attacks per unit time for each bat using a Poisson generalized linear mixed model (GLMM), with the contexts (i.e., single/multiple) treated as a fixed effect (see Section 2.4 for details). The exit rate was defined as the inverse of the mean seconds of patch residence time of bats at the foraging site.

Because no significant day-to-day differences were detected in inter-arrival intervals or the patch residence time (all *p* > 0.05, Kolmogorov–Smirnov tests), these rates were estimated using pooled data from all three days. In contrast, inter-attack intervals differed significantly among days (*p* < 0.001, Kolmogorov–Smirnov test), and therefore day-specific attack rates were used in the model. Note that, since weather conditions were cloudy throughout the three measurement days, weather was not included as a variable in the GLMM.

Differences in prey-attack rate between the single-bat and multiple-bat contexts were analyzed using a Poisson GLMM, in which the number of prey attacks per individual was treated as the response variable and patch residence time of individuals was included as an offset term. Note that overdispersion in this Poisson GLMM was assessed by examining whether the dispersion parameter, defined as Pearson’s chi-squared statistic per degree of freedom and calculated from Pearson residuals in MATLAB, exceeded 1.2 [38]. Context (single-bat vs. multiple-bat) was specified as a fixed effect, and the significance of its effect was evaluated using a Wald test. For individuals that engaged in multiple-bat context, attack events were assigned separately to the single-bat and multiple-bat contexts when the same individual flew alone before or after multiple-bat context. Individual identity and experimental date were included as random effects in the model.

Because prey insect abundance was not directly measured in the present study, we cannot completely rule out the possibility that temporal variation in resource availability influenced bat behavior. To indirectly assess this possibility, we tested whether prey-attack rates during solitary flights changed over time using a Poisson GLMM with a log link and an offset for residence time. The effect of time since the start of observation was not significant (β = 0.00106 ± 0.00327 SE, p = 0.747). The estimated rate ratio per minute was 1.001 (95% CI: 0.995–1.008), indicating that any temporal change in attack rate during the 60-min observation period was minimal. These results suggest that prey availability did not vary substantially during the observation period, although some influence of unmeasured environmental factors cannot be completely excluded.

Japanese large-footed bats have been reported to emit various types of social calls at foraging sites [39], some of which are suggested to function in repelling conspecifics [40]. Thus, social calls could potentially influence patch residence time and prey-attack rate during paired flights in the present study. To evaluate this possibility, we analyzed the prey-attack rate in paired-flight conditions after excluding cases in which social calls were observed, and compared the results with those obtained from all paired-flight conditions; both results are presented in S1 Table. Note that social calls were observed in only 5 of the 31 paired flights (16.1%). The magnitude of the reduction in prey-attack rate remained largely unchanged after excluding flights with social calls (S1 Table), suggesting that social calls likely had little effect on foraging efficiency in the present study. Therefore, all data, including flights with social calls, were included in the analyses presented in this study.

Simulations of the mathematical null model were conducted with each trial covering the same duration as the empirical measurements (i.e., 1 h). A total of 10,000 trials were performed to generate the distributions of flight patch residence time under the single-bat and multiple-bat contexts, which were then tested for differences from the empirical data. Using the outputs of the mathematical model as the null distribution, deviations of the empirical bat data from model expectations were evaluated using a parametric bootstrap test.

In this null model, arrival, prey-attack, and exit events were assumed to occur independently of interactions with conspecifics, and each event was described as an independent Poisson process. On the other hand, if exogenous factors such as temporal fluctuations in prey density were present, the frequencies of these events could also vary over time and potentially influence the residence times observed in the empirical data; therefore, such effects cannot be fully represented by the present null model. Nevertheless, even if such exogenous factors existed, they would be expected to affect both the solitary and multiple-bat conditions similarly, making it unlikely that they could account for the observed difference in residence time between the two conditions. Furthermore, as described above, the prey-attack rate did not show any significant temporal change throughout the observation period, suggesting that prey density was unlikely to have fluctuated substantially during the observations.

To examine whether individuals tended to occupy the foraging patch in the multiple-bat context, we analyzed which bat exited first during paired flights: the individual that arrived earlier or the one that arrived later. When multiple bats arrived at or exited from the foraging patch nearly simultaneously (with a time difference of less than 1 s), the order of events could not be reliably determined; such cases were therefore excluded from this analysis. To restrict the analysis to simple and interpretable situations, only two-bat conditions (i.e., paired flights) were considered. Individuals that experienced situations involving three or more bats were excluded from this analysis (observed only in the case of numerical simulation, see Results).

## Results

**Fig 2:**
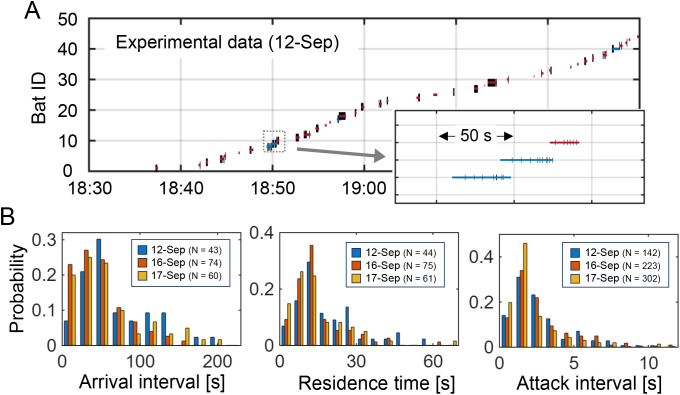
(A) Time series data of the bats foraging at the pond during 1-h measurement. The length of the line indicates the time spent at the pond. Red lines indicate the bats exiting the pond alone, whereas the blue and yellow lines show the bats exiting the pond in a group; the latter bats entered before (blue) and after (yellow) the entrance of the former bats. The vertical lines across the horizontal lines indicate the timing of attacking prey. Gray portions in the enlarged graph show the flight events of multiple-bat context. **(B)** Proportional distribution of arrival intervals (left), patch residence time (middle) and attack intervals (right) of the bats during the measurement periods for each day.

Over the three observation nights (12, 16, and 17 September), the first individual appeared at approximately 18:30 (+ approximately one hour after sunset). During the subsequent 1-h recording period, a total of 44 individuals were observed on 12 September, 75 individuals on 16 September, and 61 individuals on 17 September (Fig 2A, S1 Dataset). Bats arrived at the foraging patch successively, repeatedly attacked prey, and then exited. The inter-arrival interval, the patch residence time, and inter-attack interval averaged 57.6 ± 46.0 s, 17.3 ± 17.9 s, and 2.52 ± 2.14 s (mean ± SD), respectively, across the three days (Fig 2B). A total of 31 flight events of multiple-bat context (6, 13 and 12 events for 12, 16, and 17 September) were observed over the three days, and no cases involving three or more individuals were recorded.

**Fig 3:**
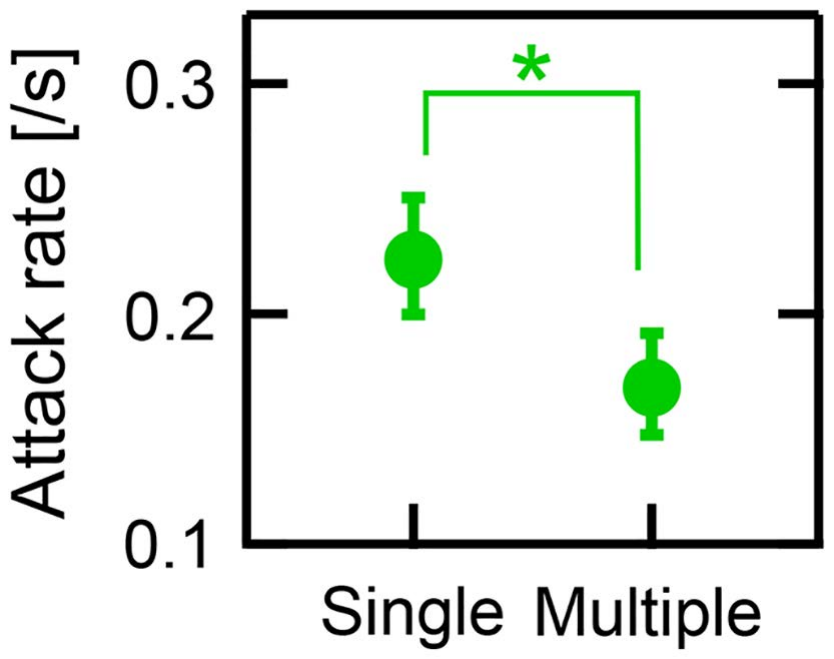
Prey attack rates during single and multiple flights estimated by a GLMM. The vertical lines show the standard errors. The slope of the fixed effect for multiple vs. single was −0.285 (p < 0.05 (*), Wald test). Number of data was 217 (single: 160, group: 57).

The Poisson GLMM of prey-attack counts indicated that bats attacked prey at a mean rate of 0.224 attacks s^−1^ in the single-bat context (estimate = −1.498 ± 0.113 SE, z = −13.21, Dispersion parameter = 0.616, *p* < 0.001), and that this rate was significantly reduced in the multiple-bat context (estimate = −0.285 ± 0.131 SE, z = −2.17, *p* < 0.05; Fig 3). Consequently, the prey-attack rate in the multiple-bat context was reduced by 25% relative to the single-bat context (exp[−0.285] = 0.75; S1 Table).

**Fig 4:**
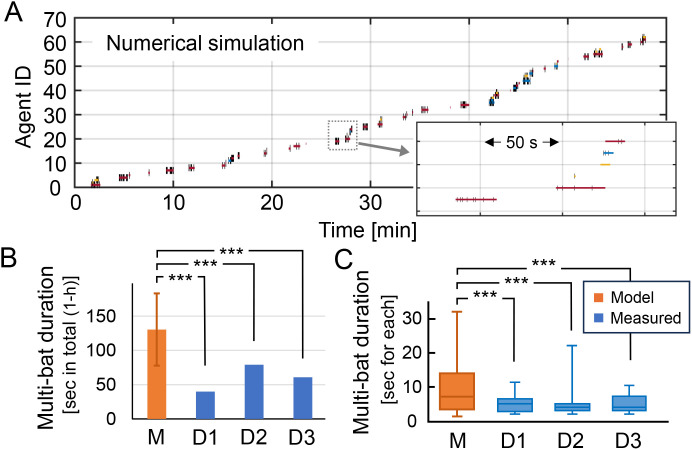
(A) Time series data of the 1-h foraging simulation of the null model (drawing rule is same as Fig 2A). **(B)** Total time spent in the foraging patch of model and measured (three different days) bats while in multiple contexts. Vertical lines on the orange bar indicate the standard deviation based on 10,000 numerical simulations. One-sample Z test was conducted; *** p < 0.001. **(C)** Boxplots (0.025, 0.25, 0.5, 0.75, and 0.975 quantiles) showing the consecutive durations of each multiple flight obtained from the numerical simulations (orange) and from three days of field observations of bats (blue). Parametric bootstrap tests were conducted; *** p < 0.001.

Using the parameters estimated from the empirical data, we conducted foraging simulations (Fig 4A). The simulated outputs—inter-arrival intervals, patch residence time, and prey-attack rates per simulation run (i.e., 1 h)—closely matched the corresponding empirical values used as model inputs (S2 Table). In the multiple-bat context, such events occurred 13.1 ± 4.0 times per hour (mean ± SD, N = 10,000 simulations), with a total duration of 130.5 ± 52.6 s per hour. In contrast, the total duration of the multiple-bat context in the empirical data was 49 s (Day 1, N = 6), 79 s (Day 2, N = 13), and 61 s (Day 3, N = 12), which was significantly shorter than predicted by the simulations (Z ≈ −2.35, p < 0.001, one-sample Z test; Fig 4B). Furthermore, the duration of individual multiple-bat contexts averaged 9.9 ± 10.0 s in the simulations (N = 131,374 events from 10,000 simulations), whereas the corresponding values in the empirical data were 5.5 ± 3.7 s (Day 1, N = 6), 6.2 ± 6.9 s (Day 2, N = 13), and 5.1 ± 3.0 s (Day 3, N = 12; mean ± SD). Parametric bootstrap tests revealed that the durations of individual multiple-bat contexts in the empirical data were significantly shorter than those predicted by the model for all days (p < 0.001; Fig 4C).

**Fig 5:**
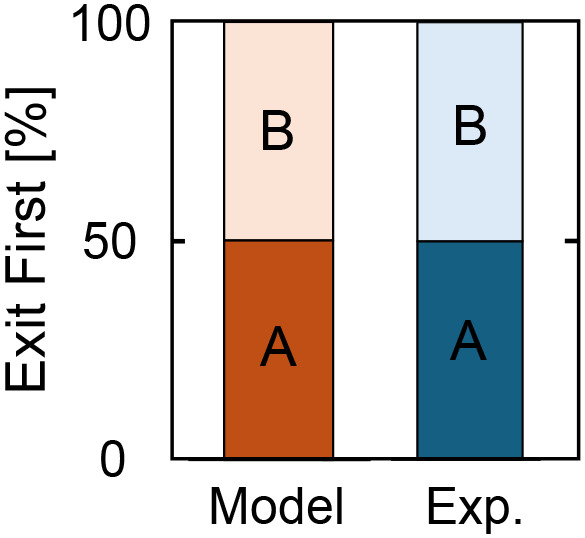
The bat that exited the pond first when in paired flights for the case of model simulation (red) and the experimental data (blue). Bat A is the bat that was staying before the paired flight together with the latter bat (Bat B). The numerical simulation was exhibited 10,000 times.

In paired flights, we examined whether the individual that exited first was the one that arrived earlier (Bat A) or the one that arrived later (Bat B). In the simulations, once a paired flight began, both bats had identical departure probabilities, resulting in an equal likelihood of Bat A or Bat B exiting first (Fig 5, left). Analysis of the empirical data revealed an exactly even split, with the first exit occurring equally often for Bat A and Bat B (Fig 5, right; N = 20; Bat A = 10 cases, Bat B = 10 cases).

## Discussion

In this study, we successfully quantified the sequential arrival, foraging, and exiting of bats within a foraging patch under natural conditions by combining a microphone array with video cameras. Our results demonstrate that when two individuals were present simultaneously within the patch, prey-attack rate was reduced relative to single-bat flight context (Fig 3). Because the pond we measured was small that flight areas overlapped during paired flights, this reduction is likely attributable to the presence of conspecifics acting as moving obstacles and/or to acoustic interference between the ultrasonic signals emitted for echolocation, which may have decreased prey-detection efficiency. Furthermore, comparison with the null model we constructed revealed that the duration of paired flights was significantly shorter in the empirical data than predicted by the model (Fig 4). This finding suggests that bats may avoid simultaneous foraging with conspecifics within a single foraging patch. The study site was located within a university experimental forest, where multiple ponds and stream pools are distributed in the surrounding area, with the nearest alternative pond located approximately 500 m from the study pond. Although we did not quantify the relative quality of these patches (e.g., insect availability or bat activity levels), bats may have moved among nearby foraging patches to reduce overlap with conspecifics.

When two individuals are co-using a foraging patch, an important question is which individual leaves first in order to avoid simultaneous foraging. Previous studies have frequently reported food patch defense behaviors, in which resident individuals displace intruders from feeding sites, in both Anna’s hummingbirds [41] and bats [42]. This pattern is generally referred to as the prior residence effect. In the present study, however, the probability that the first individual to exit was the resident or the intruder did not differ from chance levels (Fig 5), suggesting that avoidance of simultaneous patch use may not strongly depend on the order of arrival at the foraging patch. Accordingly, a clear prior residence effect does not appear to operate in the foraging behavior of Japanese large-footed bats. However, because the present observations were conducted over only three days and the number of observed events was limited, longer-term observations with larger datasets may be necessary to fully evaluate the effects of residency on foraging interactions. More generally, avoidance of simultaneous foraging with conspecifics at a feeding patch may be influenced not only by prior residence effects but also by kin selection [43], social familiarity or recognition among individuals [44], and dominance hierarchies [45]. High relatedness can promote tolerance or food sharing among individuals, whereas dominance relationships may lead to displacement or exclusion from feeding sites [46,47]. To gain a deeper understanding of the mechanisms underlying collective foraging in bats, future studies should jointly examine foraging behavior, social hierarchy, and relatedness within colonies.

Foraging efficiency during multiple-bat flights with conspecifics is likely to depend on the size and quality of the foraging patch. From the perspective of optimal foraging and patch-use theory, animals are expected to distribute themselves among feeding sites by balancing the energetic benefits of resource acquisition against the costs associated with competition and interference [1,2]. If bats can fly within a patch while experiencing minimal overlap in flight paths or sensory interference from conspecifics, the presence of other individuals may not necessarily reduce prey-attack rate and may even provide informational benefits through social cueing or eavesdropping [33]. However, as local bat density increases, interference competition and acoustic masking are expected to increase, potentially reducing foraging efficiency and promoting shifts to alternative foraging patches. Such density-dependent redistribution of predators among feeding sites is broadly consistent with the framework of the ideal free distribution [48], in which individuals are expected to distribute themselves so that foraging payoff becomes approximately equal among patches. The present results suggest that patterns of patch use were associated with prey-attack rates and co-use duration under natural conditions, providing field-based evidence relevant to the relationships among sensory ecology, collective foraging, and patch-use theory. In bat research, it has been reported that increases in bat density at a given foraging patch can lead not only to competition and subsequent shifts to alternative patches [27], but also to acoustic interference among conspecifics, which may alter echolocation behavior and acoustic repertoires [49,50]. In the present study, because multiple ponds are distributed along the river within the experimental forest where our study pond is located, Japanese large-footed bats may similarly compare prey availability between the focal pond and other nearby foraging patches and avoid paired flights in order to maintain foraging efficiency.

One important limitation of this study is that the analysis was based on recordings obtained from a single pond over only three nights. The primary reason for this limited temporal and spatial scale was the enormous amount of analysis time required to comprehensively screen all acoustic recordings collected simultaneously across multiple channels together with synchronized video data. This procedure enabled us to identify all bats visiting the pond and to distinguish individuals not only during solitary flights but also during simultaneous flights involving multiple bats, thereby allowing accurate quantification of attack frequency and residence time for each individual. To our knowledge, no previous study has comprehensively recorded and analyzed both flight behavior and echolocation calls of bats within a single pond at this level of detail. Thus, the highly detailed and individualized dataset itself represents an important aspect of the present study. In this sense, our study also serves as a pilot study for investigating patch-use behavior across different species, habitats, and seasons. Because the number of study sites and sampling nights was limited, further studies across broader temporal and spatial scales will be necessary before the findings of the present study can be generalized.

Also, because the present study was conducted over a relatively short period and within a limited seasonal window, the observed patterns may vary depending on longer-term ecological and social conditions, such as early spring conditions or the post-volancy period. In particular, seasonal differences in prey availability, energetic demands, or the proportion of inexperienced juveniles may influence simultaneous patch use and competitive interactions among individuals. Therefore, future studies applying the present framework across longer observation periods and different seasonal stages will be important for evaluating the generality of these behavioral patterns.

In conclusion, our study shows that paired patch use in *M. macrodactylus* was associated with lower prey-attack rates and shorter co-use durations than expected under the null model. By quantitatively characterizing foraging behavior that is otherwise difficult to capture under natural conditions, this study highlights the Japanese large-footed bat as a valuable model system for investigating the relationships among collective foraging behavior, patch use, and sensory interference in the wild. These findings provide a foundation for future studies incorporating multiple foraging patches and temporal variation in prey availability. In addition, understanding how bats distribute their foraging activity among nearby patches may contribute to conservation and habitat management strategies by clarifying how the spatial arrangement and connectivity of aquatic foraging habitats influence bat behavior. Such knowledge may also help evaluate how local bat density and competition affect foraging success, as well as how human-induced environmental changes, such as habitat fragmentation or the loss of small water bodies, may alter foraging dynamics within forest landscapes.

## Supporting information

S1 Dataset Times of pond entry, pond exit, and prey attacks by bats. (XLSX)

S1 Table Prey-attack rates calculated by the GLMM for each day. (DOCX)

S2 Table The three parameters calculated using experiment and simulation data. (DOCX)
