# Supplementary material for: Avoidance of simultaneous patch use in Japanese large-footed bats
Source: PLoS One. 2026 Jun 30;21(6):e0343485. doi: 10.1371/journal.pone.0343485 (PMC13318039; doi:10.1371/journal.pone.0343485)
Supplement: S1 Table — (DOCX) [file pone.0343485.s002.docx]

# Supporting information

**S1 Table**. Prey-attack rates calculated by the GLMM for each day.
