# Supplementary material for: Avoidance of simultaneous patch use in Japanese large-footed bats
Source: PLoS One. 2026 Jun 30;21(6):e0343485. doi: 10.1371/journal.pone.0343485 (PMC13318039; doi:10.1371/journal.pone.0343485)
Supplement: S2 Table — (DOCX) [file pone.0343485.s003.docx]

# Supporting information

**S2 Table.** The three parameters calculated using experiment and simulation data.

* Calculated by the GLMM (see S2 Table).

** Average of the 10,000 times calculation of number of attacks during single- and multiple-bat contexts divided by total time of single- and multiple-bat contexts, respectively.
